# At the nexus of ludology and narratology: Advances in reality-based story-driven games

**DOI:** 10.12688/f1000research.129113.1

**Published:** 2023-01-11

**Authors:** Lei Chen, David Dowling, Christopher Goetz

**Affiliations:** 1 School of Journalism and Mass Communication, University of Iowa, Iowa City, USA; 2 Department of Cinematic Art, University of Iowa, Iowa City, USA

**Keywords:** Storytelling, video games, ludology, narratology, gameplay, Detroit: Become Human, Chinese Parents

## Abstract

Story-driven games are growing in popularity across a wide range of genres. However, the narrative potential of video games is still being debated, particularly in light of the so-called tension between gameplay and storytelling. This study proposes that rules and game mechanics perform narrative semiotic functions, offering a ludic grammar of interactive storytelling. Case studies of four representative games show through exploratory player action shaped by rules, the medium of video games can generate meanings that traditional media cannot, thereby better achieving their narrative goals.

## Introduction

The last decade has seen a surge of creative experimentation in video game storytelling granting unprecedented agency to audiences of reality-based storytelling. Digital games increasingly combine cinematic perceptual realism with a modular flexibility and immediacy that exceeds even gamebooks as a medium. Although *Detroit: Become Human* (2018) has generated a great deal of popular online commentary as well as scholarly interest, the assumption that visually immersive story worlds hold the key to narrative power in the space of video games has been effectively dispelled, especially in the cases of non-action, hand-illustrated text box games *Closed Hands* (2021); *Bury Me, My Love* (2017); and *Chinese Parents* (2018) *.*

However, in the opinion of some researchers, such as Ian Bogost's, video games have still not proven themselves to be a successful storytelling medium, since even if games can tell stories well, film, television, and literature all tell them better ( Bogost 2017). As Bogost (2017) points out, stories told by walking simulator games, like *What Remains of Edith Finch*, can also be experienced as a traditional time-based narrative, such as movie, since they are entirely linear, and interacting with the environment only gets in the way. When players often cannot find the location of the next scene that contains narrative content. In his view, rather than attempting to tell stories capable of the narrative power associated with cinema and literature, video games are more adept at generating meaning through player actions, particularly by offering independent exploration of virtual worlds and systems within a defined set of rules ( Bogost 2017).

The purpose of this paper is to reveal that while it is true that poorly designed games can cause game mechanics to hinder the story experience, the tension between the two is not inevitable. In fact, game mechanics are essentially symbiotic with narrative. We demonstrate precisely how ludic elements, such as rules, interfaces, and technology of the text box, have become the core game mechanic sparking the latest advances in video game storytelling.

Another essential argument this essay pursues in each case study is that the supposed opposition between ludology and narratology proves illusory, especially when play itself is figured as an act of reading. Reading a game is a form of immersion in a reality-based character’s situation that demands critical understanding and interpretation. While choices in some games can be made haphazardly or even at random, making an *informed* choice involves players in a series of expected and unexpected outcomes. Informed choices grow in significance as consequences accumulate in what amounts to the co-construction of the game’s narrative and its meanings. We suggest that the rich possibilities of meaning are most interesting in nonfiction games, which explicitly address and seek to comment upon explicit, real-world phenomena.

Thus, the four games examined in this study are reality-based. Their stories engage seriously with social, political, and psychological topics. Namely the non-fiction topics informing these case studies are American slavery ( *Detroit: Become Human*), the Syrian refugee crisis ( *Bury Me, My Love*), the 2017 Manchester, England terrorist bombing ( *Closed Hands*), and the collective memory of childrearing in China during the 1980s and 1990s ( *Chinese Parents*). These games combine earnest and intimate engagement with political topics of global concern with especially ambitious and complex writing. Further, these games are all developed outside the U.S.—the first two are French, followed by British and Chinese titles—highlights the global reach of video game storytelling. Our reading of these games interrogates the apparent correlation between the ethical treatment of reality-based topics and interactive storytelling as a game mechanic. The broader contexts of these games cast questions about player agency (and choice) in a new light.

## Theorizing ludic narrativity

Our study builds on Green’s (2017) revelation that “sharp divides across ludological and narratological lines must be erased, for the larger benefit of both avenues of video game exploration” (14).

Although Frasca (2003) has claimed that the ludologist vs. narratologist debate never existed since “ludologists love stories too,” his earlier work proposes the dichotomy of simulation versus narrative and suggests that the true prowess of the medium of the video game lies in its performance of the former. Frasca (2001) indeed is responsible for contributing to the early definition of ludology as the study of games, which he defined in opposition to literary approaches, underscoring that “games cannot be understood through theories derived from narrative.” Ludology thus came to be understood in the early 2000s as the study of gameplay and game structure as defined by rules and restrictions for player interaction. Juul (2001) adds that narratives are often confused with video games because the player can *retell* the story of their game session so that narrative sequence (like the reward of a cut scene) can be generated *through* (yet is not equivalent with) gameplay. Several structural traits are shared by video games and narratives. Crucially, ludologists and narratologists may be operating according to disparate definitions of narrative, with the former understanding storytelling as a fixed causal chain of events that are recounted and/or reenacted, whereas the latter sees narrative “as a loose fictional frame” applied to a variety of experiential phenomena ( Egenfeldt-Nielsen *et al.* 2020, 226; Ryan 2004; Grodal 2003). Early thinking in ludology views video games as a “story-generating systems” akin to real life—that is, as “primary, real-time phenomena” ( Aarseth 2004, 50). Narrative, on the other hand, is seen as a “secondary” phenomenon, as “a revision of the primary event” (50). Such a view positions games alongside “primary,” real-world phenomena, and seems to automatically foreclose the possibility of a strong *secondary* relationship (e.g., between a game and a serious, real-world issue that it comments upon). The early ludology approach thus seems more appropriate for games with fictional frames and interests that seem to swerve away from reality and are instead rooted in fantasy and empowerment. In contrast, the games at the heart of this study are understood as profoundly related to the real-world issues they explore. And the text box itself—emblematic of narrative in games more generally—serves as both a mechanical reworking of real-world issues (their organization into key decision point, as a perhaps “secondary” framing of a primary event) and as the point of entry for the player’s own significant involvement in these issues.

Notably, the ethics narratives of our cases represent the digital remediation of print culture’s Choose Your Own Adventure stories first published by Chooseco (which sued Netflix for infringing upon its trademark in the aforementioned interactive film *Bandersnatch*) in the 1970s. Crucially, the Choose Your Own Adventure gamebooks declined in popularity with the rise of narrative video games from the late 1980s through the 1990s. As Jamison (2022) notes, by the mid-1990s, the novel experience of controlling the narrative you are inside of “felt more seductive in digital media—where the worlds were more visually immersive, and the choices more constant.” Choices in video games also encompass a much wider range of narrative affordances and actions, from the inconsequential to the existentially significant, than printed gamebooks. The books were discontinued in 1999, due in large part to the capacity of video games to tell this type of story in a much more powerful register, one capable of addressing enduring political and social questions in serious, reality-based areas such as race and immigration. Janet Murray (1997) famously ponders who will be the first Shakespeare of the medium, a “cyberbard” capable of bending the technology to more artistic effects (71). Given the evolutionary trajectory of the medium, however, we are more likely to encounter a critical historian like Thucydides than a Shakespeare. The master storytellers of the medium have begun to emerge not in the realm of escapist fictional content, but in reality-based video games on major social and political topics, narratives that unfold on epic scales with all the rhetorical techniques of fiction. Nonfictional subjects previously reserved for historians and journalists are now in the hands of game developers.

This movement in game development breaks down the binary between ludology and narratology anticipated by Frasca’s (2003) own recanting of his previously firmer stance to counter accusations of defensiveness against the so-called “foreign” colonization of game studies by literary scholars on the one hand, and a strain of essentialist radicalism on the other. He notes that “the work of the so-called ludologists does not reject narrative, nor does it want to finish narrative elements in video games,” as he is careful to point out that “accusations of radicalization of this debate are totally unfounded.” Our approach instead seeks to show empirical evidence through *the games themselves* and their mechanics for storytelling that the terms of the debate are unnecessarily locked into a false binary. Seeking overlaps in the two camps through the oblique concessions of ludologists to narrativity, may provide a starting point, but is essentially a misplaced endeavor that elides the need for discussion of actual games and how they function to produce stories.

Our approach thus resonates with Green’s (2017) point that privileging ludology over narrative inappropriately “reduces video game study to an “either/or” scenario, such that one *either* considers only the mechanics of a game *or* examines story in the absence of acknowledging the confluence of technology, game, and player” (13). For games without a clear ending that decides a winner, narrative is not rendered subordinate to skill grinding to attain points, levels and badges. A less acquisitive model, such as that showcased in *Walden: A Game* (2017), is indeed the intent for *Detroit: Become Human.* The player’s quest in each game is for the extraction of meaning through interaction with the virtual world. It should be noted that, whereas ludologists such as Bogost (2017) would categorize *Walden: A Game* as a “walking simulator” that “devolves into conceptualism,” *Detroit: Become Human* showcases action scenes featuring police conflicts and dramatic hostage situations within a science fictional setting more common to video games. *Detroit: Become Human* indeed bears all the trappings of a first-person shooter dystopian action game, only with a choice-driven game mechanic in place of combat as the chief mode of gameplay. The humble and underappreciated text box, along with associated devices of the dialogue wheel, and interactive fiction, constitutes the engine behind not only the storytelling prowess of Cage’s magnum opus, but a wave of indie PC games that transform the *Choose Your Own Adventure* story into a visceral, dynamic form of play fraught with risk, consequence, and temporal pressure. In the cases selected for this study, winning in the traditional sense is not the goal; pursuing a viable life course through a set of existentially important decisions with radically different outcomes instead transforms the player into an author in games granting agency. In non-choice games with less agency, prescribed narrative is performed according to a more linear, cinematic model. All of the cases examined here represent the ways in which the development of branching narratives entails a labor intensive creative process that breathes new life into this still-emerging medium. Building branching narratives in digital games is “an often underappreciated effort,” according to the developer of the PC game Zenuel: “You wouldn’t expect text in a game to be so divisive, or so complex, but when done right, it empowers every area of a game, enriches even the smallest details and makes a world feel like it knows you’re there” ( Nelson 2018).

This form of gameplay mechanic, which we call *ludic narrativity*, is not to be confused with another type of game featuring unstructured play in an open world, or sandbox. The narrative form associated with sandbox games has been described as *the complete graph structure*, which grants the player total freedom to navigate along bidirectional paths with all nodes linking to each other ( Ryan 2001, 247). A coherent narrative is impossible to guarantee according to this form since the player has full control of action and movement. Instead, in our cases the player follows a series of prompts with multiple outcomes, whereby they navigate their own course through a variety of branching paths. Our cases represent a range of player agency directly correlated to the number of available paths toward different outcomes. *Bury Me, My Love* offers the least amount of player agency, yet as we explain in its case study, the unilinear, chronological progression of the game—a form akin to sightseeing with a tour guide—is complicated by its multiple distinct vectors with brief side branches, each of which effectively showcases the weight of its real-world subject of the Syrian refugee crisis.

Scholarly discussion of narrative in video games has addressed the problem of allowing players to act freely, while ensuring their actions produce an interesting story. Management of this tension is a key reason for the larger scale of interactive writing, as seen in the roughly 130,000 words that constitute the narrative architecture of *Closed Hands*, a volume far greater than most novels. This vast canvas for storytelling is an extension of expansive digital age storytelling particularly evident with the advent of streaming video on demand (SVOD), that gave rise to binge-watching, and now with podcasting, binge-listening, as mainstream forms of media consumption. The sheer volume of content speaks to the encyclopedic property of interactive storytelling, which is enabled by the data storage capacity of computers, as Murray (1997) observes in *Hamlet on the Holodeck*, her foundational study highlighting the literary potential for storytelling through emerging technology. Endowed with such a massive capacity for data compared to traditional media forms designed to deliver narratives, spatial storytelling is enabled by the power of computers to represent navigable 3D space. With encyclopedic properties and spatial storytelling, video games can offer either a narrow or wide range of choices to players, depending on the developer’s aims.

If agency is relatively limited to a narrow range of choices with little impact on the shape of the narrative, the game’s storytelling becomes more performative, as in the case of *Bury Me, My Love.* Although the game contains 20 endings, the player has little agency in determining the course of each of those narrative lines. Indeed, some players of *Bury Me, My Love* complained of not having enough agency, showing that the game can be about performance rather than a choice structure. Nonetheless, the ludic quality of each of those narrative threads, as the case study will later demonstrate, suggests that narrativity is the primary game mechanic, although more like advancing pages in a novel (through the game’s simulated WhatsApp interface) rather than steering the outcome of a Hollywood screenplay. Micro choices in dialogue still funnel back to the main channel of the narrative, holding players to the course of the fixed outcome, a kind of virtual jungle cruise with a few minor diversions along the way, but never enough to deviate from the inevitable flow of experience. Murray’s (1997) concept of interactive storytelling posits far greater agency than this game affords, one that envisions an elaborate choice structure like those of *Detroit: Become Human*, *Closed Hands*, and *Chinese Parents.* The participatory properties integral to interactive storytelling are defined as the digital medium’s technological capacity to influence the production of the player’s behavior when engaged with the game. Procedural properties constitute the game’s capacity to execute rule in succession to generate behavior ( Murray 1997).

The latter point regarding rules has been emphasized by ludologists in support of the concept of procedural rhetoric, which Bogost (2017) has touted as the core function of video games’ capacity to make meaning in a more substantial way than a gratuitous cut scene or superficial story to justify the strategy and action of gameplay (not unlike the largely irrelevant war signified in a chess match). However, our case studies will demonstrate that procedural properties and the game mechanic itself are vital to narrativity via the participatory properties. Despite the relative lack of agency in *Bury Me, My Love*, for example, players must advance the game themselves and must “interact” with the main character Nour—through small, sometimes quotidian, yet deeply humanizing simulated WhatsApp texts that advance the larger narrative—who is escaping Syria to seek asylum in Europe. This question of agency and freedom of player choice, or lack thereof, bears important implications for authorship to be discussed in each case. We contend that literacy is the key to gameplay in story-driven games, as our cases suggest the range of expression possible across various genres developed specifically to honor not only visual literacy, but the multiple forms of literacy (including spatial, kinetic, audio, and social media tools) in the twenty-first century that render narrative import through digital media.

This study therefore takes it as axiomatic that game mechanics, rules, and procedural properties, while often distinct from narrative content, can function as the engine driving interactive storytelling. The following case studies show how ludology is hardly distinct from narratology, but rather one and the same, inextricably bound in a mutually reinforcing relationship. The aim here is to complicate firm distinctions in formulations of games as constituting what Mayra (2008) describes a variety of “similarly interactive systems, with a specific emphasis on meaning-making through player action (ludosis), as contrasted with meaning-making as decoding of messages or media representations (semiosis), typical for such cultural systems as television show or contemporary poetry” (19). The diverse range of forms that video games can take indeed distinguishes them from television and contemporary poetry. Many of these forms exhibited in our four chosen games reveal ludosis, or how player action—mainly through text box choices uniquely contextualized and expressed through distinct graphic aesthetics and temporally adjusted interfaces—provides the kinetic agency of meaning-making as an act of decoding the media message (in this case narrative content). But the meaning is not in the tale, but in the telling; or as Marshall McLuhan said, “the medium is the message.” Gameplay for these and other story-driven games is tantamount to unraveling the narrative yarn. Studying narrative without examining the means by which they are put into play strips the game of its ludosis, just as examining game mechanics without reference to its story, particularly for narratively ambitious works such as *Detroit: Become Human*, denies its semiosis. As Herman and Vervaeck (2005) note, “the way in which a story is narrated … turns it into what it is.” He explains, “those who insist on denying the importance of the method of narration by reducing a story to content might just as well go to the movies or watch television because both of them can offer similar content” (7).

## Methods of analysis

Purposive sampling led us to our information-rich case studies suitable for qualitative research in digital media such as this interpretive critical analysis of ludic narrativity in video games. It aims to achieve not total coverage or generalizability according to quantitative randomized sample studies, but “a deep, albeit partial and contingent, understanding of social reality in a specific context” ( Lindlof and Taylor 2019, 143). Our sample is thus illustrative rather than definitive. In order to examine the leading edge of the genre, we selected four reality-based story-driven games that use ludic elements like text/dialogue boxes as the primary game mechanic that advances their narratives. The games were chosen to represent the various sectors of the global games industry at the leading edge of narrative video games: *Detroit: Become Human* by the French game developer David Cage and Quantic Dream (2017), *Bury Me, My Love* also by a French game developer Florent Maurin and Pixel Hunt (2017), *Closed Hands* by the British developer Dan Hett and PASSENGER (2021), and *Chinese Parents* by the Chinese developer Moyuwan Games (2018). These critically acclaimed games are all reality-based and employ distinct game mechanics to deliver complex narratives treating serious issues of learned debate with contemporary and historical significance. *Detroit: Become Human* is an allegory for U.S. slavery and the Civil Rights Movement (which anticipates the recent BLM protest movement); the Syrian refugee crisis is the focus of *Bury Me, My Love*; international terrorism constitutes the subject of *Closed Hands*; and *Chinese Parents* centers on Chinese collective memory of child-rearing from the perspective of parents.

We selected games featuring narratives bearing on social and political reality to indicate that a strong correlation exists between technical innovation in video game storytelling and the real-world relevance of their chosen topics. Although our case studies consist of titles that are technically designated as fiction, they are committed to social reality just as “a theatrical play may be documentary as easily as it is fiction,” as Aarseth notes (2016, 491). “For representational games,” he explains, “this means that as long as the game objects refer to events and existents in our world (e.g., in our history), they do not fictionalize but document.” The realistic import of these narratives heightens their urgency—as well as the precision of the affective and documentary nature of their telling—given their applicability to current political circumstances. We thus approach the fictional games of our case studies as bearing documentary functions with important sociopolitical implications, according to the formulation that “documentary simulations and games do not have to refer to specific objects to be documentary; they can also refer to generic objects (e.g., a type of airplane) and the resulting simulation is still documentary, not fictional” ( Aarseth 2016, 491; Fullerton 2008).

Narrative ambition unites our selected story-driven games, as reflected in the multiple endings possible in each, with six outcomes in *Detroit: Become Human*, 20 in *Bury Me*, *My Love*, nine in *Closed Hands*, and 100 in *Chinese Parents.* The rationale for our selection process prioritized games representing self-conscious attempts at achieving sophisticated narratives from across the global games industry, from mainstream AAA to indie and fine arts sectors. The various approaches to narratives in our sample thus showcase the adaptability of finely crafted narrative to different conventions and technological affordances. Originally released on PS4 and Microsoft Windows, *Detroit: Become Human* represents narrative innovation in the console game sector. The indie mobile game market’s foray into story-driven games appears in *Bury Me*, *My Love*, which is designed to be played on a smartphone, yet in an immersive way demanding extended engagement. As a conceptual game from the world of digital fine art, *Closed Hands* was designed for the PC specifically with use of headphones to enhance the game’s polished sound design carefully attuned to the nuances of the story. Whereas *Bury Me, My Love* and *Closed Hands* cater to niche audiences, *Chinese Parents* is a mass market production, which has won considerable market share in the highly competitive Chinese video game industry, particularly the casual indie RPG simulation sector. Thus diversity is represented in the sample in terms of aesthetics and gameplay as shaped by each title’s cultural, geographical, and industrial situatedness.

The multilinear and interactive nature of story-driven video games poses a challenge for narrative analysis ( Plewe and Fursich 2018, 2473). Our method of critical analysis derives from approaches recommended by games scholars. With a focus on interface, rules, and goals, one approach examines entity manipulation ( Zagal *et al.* 2005). A more holistic method considers not only formal and technical aspects, but also cultural and semiotic concerns ( Konzack 2002). Our study’s focus on how technical aspects—figured according to ludology’s concept of procedural rhetoric—are inextricably bound to narrativity suggested the use of Carr’s (2009) method. Her approach draws from Barthes’s structural and textual method to recommend analyzing games as “playable text” at the levels of both rules and game mechanics as well as narrative content ( Carr 2009, 1-2). We build on this method by proposing that rules and game mechanics perform narrative semiotic functions, offering a ludic grammar of interactive storytelling. That is, texts are not only playable according to Carr’s (2009) formulation, but from the reverse perspective, we posit that rules and game mechanics can be seen as textual insofar as they generate narrative through the player’s choices. Unlike Plewe and Fursich (2018), whose case study methodology separates analysis of rules and game mechanics from narrative content under the assumption that they are discrete entities, we combine treatment for each case. We posit that the game mechanic is the technological tool through which players access and engage the language of the game’s narrative, giving it shape through their own interactions.

Games were examined for 1) synchronization of gameplay mechanic with narrative development, 2) formation of narrative structure, 3) thematic coherence, and 4) their impact on player experience. Additionally, paratextual promotional videos, playthroughs, reviews, and developer interviews and conference presentations were also accessed to contextualize the analytical process. Critical interpretation of formal aspects of the games is drawn from Aarseth (2003) and Zagal *et al.* (2005), particularly in terms of rules and game mechanics. Such technical features are vital to narrative tone and import, forming the empirical evidence to demonstrate the games’ *ludic narrativity*, defined as gameplay that tells a story through “units of meaning, or semes” ( Carr 2009, 5). Player action, we argue, is the narrative glue that makes the disparate semes of the game cohere with what Carr (2009) calls “a binding quality,” one that forms “clusters and continuities with, across and beyond text” (5).

## The (in)visible work of *Detroit: Become Human*

*Detroit: Become Human* developer David Cage described writing the text for the game as a painstaking process of conceiving of its many different actions, consequences, and outcomes. Interactive writing, he noted, entails consideration of not only space and time, but also possibility. Each situation demands imagining thousands of variables, conditions, and options. Whereas film scripts typically contain roughly 100 pages, interactive stories like that of *Detroit: Become Human* can contain between 4-5,000 pages. Such sprawling narrative landscapes are essential to generating a plausible sense of player agency. In conscripting the player in the authorial role, gameplay becomes tantamount to an act of creative writing. Cage (2018) pointed out that the medium awaits the arrival of its own Stanley Kubrick or Orson Welles, because “Interactive storytelling can be what cinema was in the twentieth century—an art that deeply changes its time.” The goal for video games, as envisioned by Cage (2018), is to achieve the power and range of narrative expression previously associated with literature and film. Ian Bogost (2017) argued that such a goal is pointless for video games and anathema to their nature. In his view developers should instead focus on rendering subjects visible, operable, and perhaps even beautiful through gameplay. *Detroit: Become Human* thus defies Bogost’s (2017) assertions that “the future of games will be one in which games abandon the dream of becoming narrative,” and “to use games to tell stories is a fine goal, but it’s also an unambitious one.” The culprit, he claims, are walking simulators like *What Remains of Edith Finch*, a narrative masquerading as a game, which he believes would have been better told as an animated film.

Cage’s (2018) invocation of cinema as a standard of excellence defining the achievement of the medium’s maturity came shortly after—and arguably in response to— Bogost’s (2017) lament of “cinema envy” in the game industry. Story-driven games present a wrong-headed evolutionary trajectory of the medium, one that plays to another medium’s strengths while neglecting its own, particularly game mechanics and player choice, according to Bogost (2017). He decries this segment of “the game industry that has long dreamed of overtaking Hollywood to become ‘the medium of the twenty-first century,’ a concept now so retrograde that it could only satisfy an occupant of the twentieth.” *Detroit: Become Human*, however, uses the player’s choices to determine the outcome of six different endings. The game’s ostensible subject is its narrative, which gameplay indeed renders “visible, operable, and beautiful,” a process whereby storytelling itself on behalf of player choice is simultaneously ludic and semic ( Bogost 2017). The game does not sacrifice action for contemplation. Choices are freighted with immense ethical pressure, which is intensified through the use of a time bar forcing the decision to be executed within seconds. The effect is to dramatize the difficulty of each decision, one that compounds and complicates the radical agency the game grants to players, who can precipitate war, peace, or go rogue. That intensity is compounded by the real-world implications of the plot.

As androids emerge into sentience, they become increasingly rebellious and strategic in their quest for freedom. Awareness of one’s own oppression as a necessary precondition to escape from slave labor echoes the pattern of Frederick Douglass’s liberation in *Narrative of the Life of an American Slave.* In it, Douglass details his enslaved childhood in the antebellum South where his captors systematically denied slaves literacy—and thus access to abolitionist writings—as a means of quelling rebellion. Once Douglass successfully teaches himself to read, he accesses abolitionist writings that enable him to comprehend the full extent of his oppression. This pattern appears in the game, which emphasizes the importance of sentience as the foundation of android empowerment. As with Douglass, androids’ awareness of the limits to their rights and freedoms fuels their escape and political activism. The gameplay dramatizes this gradually evolving awareness through a number of narrative choices that move the character through the storyworld by entering their actions on the controller. The desire to be free throughout the game is not abstract, but a response to a specific set of material conditions.

Significantly, player action occurs not as a question of self-indulgent personal preference, as with the gleeful anarchy and radical individualism of characters in *Grand Theft Auto V*, but with reference to the action’s impact on the larger android struggle for freedom. Gameplay in *Detroit: Become Human* therefore significantly departs from the narcissistic quest for self-aggrandizement and elevated social status driving games such as *Fortnite*, as seen in the quest for skins and character accoutrement that gratuitously capitalize on personal brand amplification endemic to digital culture and self-stylization ( Dowling 2021, 94). Instead of leveraging player choice as an acquisitive form of consumer capitalism whereby skill is rewarded with faux capital via points that are visually valorized through badges, loot, and coveted skins, player choice in *Detroit: Become Human* is deliberately politicized. Whereas the path forward to liberation and equal rights appears in each player choice as a complex ethical dilemma—which presents a sophisticated understanding of how this and other social movements take shape through real decisions in isolated moments—the game neglects such careful consideration of why particular groups of humans have been (and continue to be) disqualified from the rights and freedoms enjoyed by others ( Schubert 2021). As signaled in the title, the game’s forward-looking focus is on the challenge of becoming human, providing a meditation on the influence of individual choice on racial justice in the U.S. Nothing less than the fate of an oppressed minority rides on player choices, each of which are situational, temporally limited, and chart a unique narrative course.

Agency is central to *Detroit: Become Human*’s gameplay mechanic. Whereas players must have quick reactions in physical conflicts and for complex tasks, their more ethically laden decisions that directly shape the narrative occur through text box options that allow the player to choose what to say or do. As Schubert (2021) observes, “narrative and gameplay in *Detroit: Become Human* are tightly interwoven and hard to separate.” The game’s mechanics thus catalyze “a larger, interconnected system by which a digital story takes shape” ( Green 2017, 12). For example, the gathering of evidence for “The Hostage” chapter that opens the game conscripts the player in the role of co-author of the narrative. The nature of the evidence gathered by the player dictates the outcome of the chapter, precisely because that data determines Connor’s approach to rescuing a hostage taken by a deviant android. The game mechanic does not allow the player to overpower the deviant android, but instead requires the player to interpret the evidence available in the storyworld to achieve a safe resolution. As Schubert (2021) notes, “gameplay decisions in *Detroit: Become Human* are (almost always also) narrative decisions” (12). Quicktime events and dialogue choices extend the narrative branches. The branching story co-authored through gameplay is displayed at the end of each chapter. This detail underscores the importance of narrative as the main accomplishment or product of gameplay, which is constructed as a semantic exercise in critical thinking and interpretation of the simulated environment.

The individualism of a traditional first-person shooter, as in battle royale games like *Fortnite*, ends in the death of the player’s avatar. By contrast, the death of the player’s avatar in *Detroit: Become Human* does not end the game, as play may continue to support the plight of androids through the two other playable characters. Narrative construction is thus showcased as the core value of gameplay, as Cage and his designers preserve the player-generated story by continuing, rather than erasing, progress upon a protagonist’s death. The ludic narrativity of android liberation can thus be constructed through multiple protagonists.

Player investment in shaping the through line of the unfolding story of *Detroit: Become Human* is reinforced through flowcharts which appear at the end of each chapter. The flowcharts allow characters to rewind certain scenes to either recant decisions or lament them. This detail positions gameplay as a form of authorship, not in the abstract, but in the literal telling of the fate of the liberation movement. Conner’s character is not locked into his programming to suppress the liberation; he may deviate from it to aid the uprising, but can also abruptly decide to shoot Markus at his speech during the final android protest. Many chapters contain six different endings. The flowcharts following each chapter highlight not only the decisions players made, but also those they did not. The otherwise invisible work of interactive writing from the designer’s perspective becomes entirely visible to the player, who is invited into this dimension of the process of game production. In the process of gaining new awareness of interactive narrative production from the scriptwriter’s perspective, player consumption is enriched through a new self-awareness of their own choice pattern whereby they might elect to pursue different paths on the next playthrough. The narrative blueprint’s appearance at these key intervals echoes the flowcharts frequently used by novelists and narrative nonfiction writers. A plot chart similarly plays a prominent role in *Closed Hands*, a game in which the player becomes acutely aware of unchosen paths and their alternative consequences. *Detroit: Become Human* thus epitomizes the principle of gameplay that entails revealing consequences of actions to players within the context of the storyworld. As Murray (1997) explains, agency is “satisfying” when we “see the results of our own decisions and choices” (126).

## The performative narratives of *Bury, Me My Love*

The parasocial “trying on” of alternative identities can raise player awareness and empathy for disadvantaged and systematically oppressed populations. As shown in *Detroit: Become Human*, identification with marginalized game characters can become what Leach and Dehnert (2020) call “a means of exploring the social locations and experiences of marginalized others,” especially in the construction of racial identity and strong female protagonists (23). *Bury Me, My Love* (which translates from *take care; you better not predecease me* in Arabic) similarly immerses players into the experience of a marginalized population, particularly through the reality-based journey of a Syrian refugee migrant. Rather than recreating a visually ornate and elaborate storyworld to simulate that experience, Florent Maurin and his team of developers of Pixel Hunt designed the game from the perspective of the migrant’s loved one, as told through this digital correspondence in a recreated version of a WhatsApp interface. Players are recommended to experience the game on their phones to better approximate the communication between the migrant woman Nour and her husband Majd at home. That correspondence takes on a wide range of subjects and tones, from playful and intimate to serious and strategic depending on Nour’s situation along her journey. The result is a pattern of gameplay that advances the narrative via the communication with Nour, who faces desperate and life-threatening circumstances that the player must guide her through from the perspective of Majd.

Thus traditional gameplay is undermined on several fronts in *Bury Me, My Love*, each of which contributes to a unique form of ludic narrativity. Majd in this game can be construed as the player’s avatar, as the player guides Nour to safety through him and all gameplay choices dictate the content of his communication. The effects of player choices, according to Murray’s principle of best practice for narrative-driven games mentioned earlier, are immediately apparent in how they shape Nour’s condition. Majd—and thus the player—is tasked with a challenge fraught with violence and mortal risk that the developers conscientiously elected to portray off scene, or at least metonymically through the WhatsApp exchanges that take place through text box options. In addition to player-avatar relations to enhance the parasocial functions of the narrative, *Bury Me, My Love*’s adopted medium of the mobile games is not typically associated with immersive, story-driven titles. A deliberately non-visual interface for scene construction paradoxically heightens the emotional intensity of communication choices that constitute the gameplay mechanic.

The reality-based ludic narrativity of *Bury Me, My Love* was originally inspired by Lucie Soullier’s *Le Monde* article, “The Journey of a Syrian Migrant as Told by her *WhatsApp* Messages.” An experience told through a record of social media correspondence is impressive in print, but takes on an entirely new feel when such messages are activated on one’s own phone in the role of Majd. The character and player thus become one through the correspondence with Nour. As developer Maurin (2021) noted, “using interfaces people are already used to is powerful,” particularly in terms of its temporality, which is manipulated “to create a close bond between characters.” This form of gameplay that places the player in such close proximity to events crafted to replicate those of the real immigrant experience carries the potential of becoming emotionally overwhelming. Given the gravity of the content in Soullier’s nonfiction report, for example, the team of developers at Pixel Hunt chose not to include the topic of rape in the game, but elected to include Nour’s death in several of the game’s twenty different endings. According to Maurin (2021), drawing many of the twenty narrative threads to the passing of Nour was a decision made out of respect, which is depicted through audio clips that mark the end of the playthrough. “She’s fictional,” he explained, “but represents the real lives that are being lost” ( Maurin 2021). Such serious content is uncommon—if not unprecedented—in the mobile games industry, which leans instead toward more casual content designed for diversion. In this case, the mobile game highlights the use of the smartphone in the migrant experience as the lifeline to many refugees’ relationships and a key tool enabling their passage to safety.

Although Nour’s survival depends on the narrative path assigned to the player, no combination of choices made by the player has a bearing on the outcome. The gameplay leading to twenty possible endings of *Bury Me, My Love* is therefore pre-scripted. “The player has no agency,” according to Maurin (2021). “Everything is decided by hidden variables,” he explained, unapologetically adding that “some players complain that they don’t have enough agency.” Indeed, most of the choices in the text boxes may shape the tone of the correspondence with Nour in any given scene, but they in all cases do not determine the outcome. Maurin’s rationale for this design is premised on the notion that story-driven games can be performative rather than choice driven. The real-world significance of the topic led Maurin to strip agency from the game, because the question of winning is irrelevant in his view. The forced conclusions metaphorically convey the lack of agency faced by migrants. “Some immigrants do succeed and others don’t,” he urged, noting that the game raises the questions, “whose stories get told? Who is allowed to tell these stories?” ( Maurin 2021). Narrative thus appears as performative and pre-scripted in order to focus gameplay on the immediate characters as a window into the larger Syrian refugee crisis. This lack of agency should not be viewed as a limitation or liability, but rather one of the game’s many innovations described above, which include: 1) a powerful female Arab protagonist, 2) immersive, story-driven, serious, reality-based content for a mobile game, 3) violent content (via text and audio) that is not visually depicted 3) a simulated social media interface for the gameplay mechanic, and 4) a narrative structure consisting of twenty separate vectors (with brief side branches) that progress chronologically.

## Closed Hands

*Bury Me, My Love* shares with Dan Hett’s *Closed Hands* the overarching goal of raising player consciousness about the personal impact of political violence. Unlike *Bury Me, My Love*’s mobile design and performative narrativity with limited agency, *Closed Hands* offers radical player agency in shaping the selected protagonist’s narrative. Character fates do not play out on separate vectors, but can be intertwined based on player choice, predominantly through text and dialog boxes. As with *Detroit, Become Human*, player agency is positioned as an interpretive act demanding thoughtful and conscientious literacy from the player. Developer Dan Hett, who lost his brother in the 2017 Manchester terrorist bombing, structured the game around the event, which is not shown visually. Players choose from one of five characters with the option of switching among them at any time, as the narrative flowchart is accessible at any moment during gameplay. Too large to fit on the screen at once, this story tree of the game’s intricate plot options can be explored by clicking and dragging to unseen portions of it. As with the flowchart feature of *Detroit: Become Human*, this overview of the completed nodes in the plot sequence signals the effects and consequences of gameplay to the player. At the center of the flowchart is a box labeled “Incident,” a fictional terrorist bombing in a British city from which the player can select a node in the network featuring the experience of one of the characters ( Hett 2021). Gameplay incentivizes completion of each node which opens up a new, previously greyed-out, branch of the story tree. This game mechanic is figured as an act of unveiling a politically significant plot that intertwines with the other characters. One must play and complete each node to open a new thread with the goal of reaching the end of the story, upon which the player sees the message: “You’ve reached the end of a story—one of them. *Closed Hands* is an intricate story with many threads. Although you’ve reached the end of this one, others remain” ( Hett 2021). Gameplay thus proceeds with a distinctly novelistic feel whereby immersion is narrative rather than visual, as the technology never aims for graphic or audio simulation of any event.

The sense of being captivated by each of the storylines of *Closed Hands* is thus driven by the profound personal intimacy rendered in each character portrait. Sound design accentuates narrative intrigue aiding shifts in tone during any given sequence without being overbearing or melodramatic. Intimacy is achieved through scene and dialogue depicting personal, professional, and civic aspects of each character through simulations of their digital lives via instant messaging chats with co-workers and loved ones as well as menacing exchanges with threatening figures. As an alternative to featuring embodied avatars that the player moves throughout a storyworld, key aspects of characters are depicted visually through their faux digital desktops. At various stages in gameplay, the player occupies the digital desktop of each of the five main characters. Mike, a middle-aged British white male with racist inclinations associated with the Patriots group who threaten the Muslim community in the wake of the bombings, has a desktop background image featuring a giant football (soccer) stadium; Farah’s desktop background bears an official government seal reflecting her work for the Police Intelligence Service. Simulated digital interfaces provide the dramatic stage and gameplay interface for characters’ online communication. Haziq, the Muslim shop owner and father of a perpetrator of the bombing, at one point accesses his son’s computer and communicates via instant messaging with a friend of his son to ascertain his whereabouts after he had gone missing; Beth, a reporter at odds with her editor who pressures her to sensationalize the story, voice texts with him from the scene of the bombing; Markus Bashir, an eyewitness of the event who advocates for a compassionate response to the Muslim community, receives a threatening message from the Patriots that escalates into a heated correspondence on his laptop ( Hett 2021). By simulating their digital behaviors via the use of their online tools, these interfaces immerse the player deeper into the characters’ personal lives.

The greatest degree of player agency in *Closed Hands* is experienced in opportunities to select the next scene within the space of the flowchart. The choices within each scene—by contrast to the radical autonomy offered when selecting scenes and charting a unique narrative web—are essentially unilinear, locking the user into a fixed outcome. Choices in the dialogue box render the same outcome but are designed to appear different, a feature that is often thinly veiled by synonymous options like “transparency” and “honesty” in one case, and “I think so” and “I hope so” in another ( Hett 2021). Such buttons are like scrolling down in a multimedia narrative to reveal the next screen, which is essentially a form of digital page-turning. Yet scene selection on the flowchart offers opportunities to move forward or backward through time and to switch to any of the other four protagonists. The larger sweep of the narrative constructed by the player is not lost upon completion. The coda of the endings in *Closed Hands* as played from Haziq’s and Markus’s perspectives features a reflection back on the event with the reporter Beth, a detail that places the significance of the attack into perspective from the vantage point of ten years in the past for Markus.

The gameplay of *Closed Hands* generates authentic player interest and curiosity in the various perspectives and interrelated stories of these characters, one bearing heavily on the precarious position of the Muslim community in a British town following such a tragedy. Among the most poignant moments reinforcing them are Haziq’s attempt to reconcile with his son’s crime. Through Haziq, players experience the tragedy as a member of the Muslim community, which is then mediated to the general public through Beth’s reporting. Haziq identifies with Beth’s portrayal of his son Yasid as a “confused, weak boy,” a moment that brilliantly harkens back to her journalistic past as a principled idealist who defied her editors. The mainstream press’ pursuit of profit and audience supersedes concern for community voice, as she discovered early in her career when she covered political protests. Her editors thus implored her to “run it big language on the topper—bang up the drama.” In response to her insistence to wait until casualty figures could be confirmed, the editor asks “Do you wanna be the first to the story or the best but last?” ( Hett 2021). This final point suggests the keynote and overall objective of *Closed Hands* as a media product designed to defy the manic, Twitter-driven news cycle’s superficial and amplified reportage. This self-reflexive moment alluded to the game’s own slower, more nuanced process of production of an alternative interactive form of immersion into the layered, complex, and interconnected meanings of a terrorist bombing, wherein the personal bleeds into the political.

## Chinese Parents and storytelling of life course

Our last case is *Chinese Parents*, a simulation game that allows players to experience the journey from infancy towards adulthood as a Chinese child. Players have 48 turns to shape the life of their role-playing character. Their choices will affect the character’s statistics (e.g., IQ, EQ, memory, and stress level); statistics will affect the cost of skills learning, and the final statistic and skills acquired will determine what kind of life the role can have at the end. Choices need to be made in each round on how to plan the character’s schedule. Different arrangements impact the character’s statistics in different ways. For example, pushing your character to excel in academic fields will increase IQ. However, it will also add up the stress level. When under excessive stress, the character may experience serious mental issues which will result in game over. Playing Nintendo games can help relieve stress, but it will lower parents’ satisfaction. Since every choice has a trade-off, there is no easy way of gameplay, just like there is no easy mode of life, especially for new players. In addition to the player's scheduled events, there will be unexpected incidents every round, such as “being the last kid to be picked up after school.” These incidents can also affect the role’s statistics in a certain way, thus adding more unpredictability to the character’s life. After the first playthrough, the player can start a new cycle by role-playing the next generation.

In general, the game accurately depicts the life course and captures many shared experiences of Chinese children, such as working hard to meet the high expectations of their “tiger mother,” preparing hard for the ultimate challenge: Gaokao (the university entrance exam). It especially reflects the childhood of those who were born between 1980-2000. Growing up in a time that had seen an unprecedented fast economic expansion and social upheaval in Chinese history, this generation had no memories of living in abject poverty, and collectively received a good education and had relatively unrestricted access to information. Considering this, it is no surprise to see the character in the game never experience any pain of hunger or poverty. Another interesting detail is that the character has no siblings. This goes in line with the experience of those post 80s and 90s, since they are the first generation affected by the one-child policy, which limited most Chinese couples to one offspring each ( Lian 2014).

In this sense, the story told by this game is actually the story of a person’s life, or more precisely, the collective memory of a generation. By allowing players to step into the shoe of a typical Chinese child and play both the role of parents and the kid, the game gives the grown-up post 80s and 90s a chance to rethink their lives and their relationship with their parents.

This narrative goal is highly supported by the game mechanics of *Chinese Parents,* whose gameplay is heavily based on turn-based strategy. Each turn, players select a course of action from a text box that displays the available strategy options, click to execute, and the system automatically completes all actions for that turn and gives feedback. Much of the feedback is presented in the form of event summary in a pop-up text box, e.g., “Dad gets home drunk and starts shouting a large variety of well-selected curse words at you.” This event causes the character's intelligence attribute to be reduced by five. With this game mechanic, *Chinese Parents* organically integrates a large number of events that pass collective memories into the player's game experience.

This mechanism effectively increases the narrative speed, making it possible to tell the story of a growth spurt of about twenty years in about three hours. It also means that the game can have a higher narrative density, as more anecdotes can be incorporated into the story. More crucially, by adopting this mechanism, the telling of events in this game is freed from the constraints of traditional storytelling structures, which usually require the chosen events to form a high-level story structure such as a three-act structure. This is a challenge for role play games, especially for sandbox games whose plot is highly determined by players. The player may spend ten minutes doing nothing but wandering, resulting in a personal story which is far from interesting. In *Chinese parents,* the events are also not organized into a drama structure. If the game were transformed into a text, the result would be a daily stream of events with flat characters and a monotonous plot, rather than an interesting, coherent story. However, once played, it’s totally different. What a simple narrative like “Dad gets home drunk and starts shouting a large variety of well-selected curse words at you” presents is not just a daily incident but one that could result in a reduction of the character’s intelligence. The subsequent occurrence may have nothing to do with the preceding incident in reality, but it may exacerbate or mitigate the intellectual harm caused by the preceding event in the game. Events that lack connection in reality can constitute a meaningful and coherent experience for the character in the game.

This highlights a distinctive storytelling capability of video games: they are able to communicate stories that are challenging to convey through conventional narrative techniques. Many human experiences are frequently ignored because they are difficult to logically incorporate into conventional plot structures or dispersed across numerous characters. Games, with the aid of their own particular rules, can save these human experiences and bring them back to light. Therefore, when playing the game, players are not just retracing their steps but rather experiencing it in a new setting with a fresh perspective, seeing and remembering many of the things they had previously overlooked. This could prompt players to reevaluate important social issues like the relationship between parents and children, what constitutes a good education, what constitutes a successful life, etc.

## Conclusion

This study revisits well-trodden ground with a new goal in mind: to situate *reading* a game in relation to ludology vs. narratology debates. Reading as a mechanic helps reveal how ludology and narratology are mutually *reinforcing* rather than mutually exclusive categories in each of the ensuing case studies. The first of the cases considered is *Detroit: Become Human*, which represents a visually robust, action-based blockbuster noted for its narrative prowess by industry critics and media scholars ( Holl & Melzer 2021; Leach & Dehnert 2021; Schubert 2021). On the surface, the game bears the appearance of a dystopian action video game, yet its gameplay mechanics are concerned more with conscripting player agency in building narrative through ethical choices than kinetic combat as in a first-person shooter. Three text-based games comprise the next case studies, *Bury Me, My Love*; *Closed Hands*; and *Chinese Parents*, illustrating how procedural rhetoric, defined as meaning-making through player action, constitutes the engine driving each game’s narrative. These cases present strong evidence suggesting that the leading edge of story-driven games converges narrativity with player choice rather than treating them as discrete entities. Findings counter the ludologists’ charge that storytelling is anathema to video games, a view that casts narrative’s place in the medium as a facile, gratuitous window dressing superimposed on the game mechanic. The ludologists’ core premise, that the medium of video games essentially generates meaning through exploratory player action shaped by rules, can apply to the co-construction of narratives.

## Data Availability

No data are associated with this article.
