# Supplementary material for: At the nexus of ludology and narratology: Advances in reality-based story-driven games
Source: F1000Res. 2023 Jan 11;12:45. [Version 1] doi: 10.12688/f1000research.129113.1 (PMC9978239; doi:10.12688/f1000research.129113.1)
Supplement: Peer review history

# Peer review: Reviewer response for version 1

**DOI:** 10.5256/f1000research.141775.r162888

**Published:** 2023-03-14

**Authors:** Christopher Paul

**Affiliations:** 1 Seattle University, Seattle, WA, USA

The central argument of the essay is that narrative and game mechanics, or narratology and ludology, actually are better considered together rather than separately.

 The essay uses four games as core examples, including titles like *Detroit: Beyond Human* and *Chinese Parents. *Also rooted in the history of game-like aspects of storytelling as found in *Choose Your Own Adventure *books and narratives with multiple endings like the “Bandersnatch” episode of *Black Mirror*, the essay pushes against the notion that games cannot tell compelling stories.

 The authors make the case that, in video games, the game mechanics are actually a key part of storytelling than inform and alter the narrative dynamics and expand the potential of games. I agree!

 Middling questions like this and pursuing a both/and option, rather than an either/or approach is generally more appealing to me than trying to force a dichotomy and place a required choice on players and critics.

 I would also push the essay in two primary ways.

 First, I’m a big fan of the idea that games are more than just stories or game mechanics. I typically push to studying the way that words, game design, and play all work together to make meaning in games. That makes this essay interesting to me, but I also think there’s a dynamic of play and how the player interacts with the game that is not fully accounted for in this piece.

 To that end, in the repeated example used in discussion of *Chinese Parent,* the dad coming home drunk and swearing at the player is a short text prompt that changes the game mechanics but can also be made more meaningful by the interaction of that prompt and the player’s experience. It’s not just mechanics. It’s not just story. It’s the interaction of those in combination with play that help signify how meaning works in games.

 As an example, there are stories told by players in and around sports games where players develop their own narrative and story around the game in a rich interaction of imagination, storytelling, game mechanics and play. Ricky O’Donnell’s chronicling of his version of the Western Illinois basketball team or the *Football Manager *players who ply their trade in fictional countries like Morgsthia push on narratology, ludology and the middle approach of claiming both matter by underscoring the role of play and playfulness in understanding how games are made meaningful.

 Second, although I think there is plenty of room to critique Bogost’s approach and the role of procedural rhetoric in game studies, I also think this paper sometimes 'straw person’s' that argument a bit. This kind of critique of game narratives is often not that games cannot tell stories, but that they don’t tell *good* stories. With that in mind, it is hard to make the case that “Bandersnatch” is the best episode of *Black Mirror *or that *Choose Your Own Adventure *books are paragons of literary excellence. Instead, they are novel explorations of a different mode of storytelling that frequently get left in the dust by more typical approaches to narrative design, whether that be in episodes like “San Junipero” or young adult novels like *The Westing Game.*

 A primary contribution of this work is in the discussion of the case studies. The breakdown of the games is really interesting and advances my understanding of each of them. Beyond that, I love how the selected games are not necessarily ones I have already read a ton about. Pulling a diverse array of interesting games and elevating them is worthy of discussion and exploration. So, let’s move past narratology and ludology and consider games on their merits. What is interesting about them? What can we learn from them? And how can we better understand them and how they make meaning?

Is the work clearly and accurately presented and does it cite the current literature?

Partly

If applicable, is the statistical analysis and its interpretation appropriate?

Not applicable

Are all the source data underlying the results available to ensure full reproducibility?

No source data required

Is the study design appropriate and is the work technically sound?

Yes

Are the conclusions drawn adequately supported by the results?

Partly

Are sufficient details of methods and analysis provided to allow replication by others?

Partly

Reviewer Expertise:

Game studies, rhetoric

I confirm that I have read this submission and believe that I have an appropriate level of expertise to confirm that it is of an acceptable scientific standard.

# Peer review: Reviewer response for version 1

**DOI:** 10.5256/f1000research.141775.r162890

**Published:** 2023-03-01

**Authors:** Edmond Chang

**Affiliations:** 1 Ohio University, Athens, Ohio, USA

Overall, Chen, Dowling, and Goetz's research article attempts to further resolve the now decades long theoretical feud between narratology and ludology. The article argues that the two sides are in fact complementary, even supplementary through what they name as "ludic narrativity," a useful analytic that narrative is a game mechanic. I very much appreciate the survey of games-- *Detroit: Become Human*, *Bury, Me My Love*, *Closed Hands*, and *Chinese Parents*--because they offer a range of genres, platforms, topics, and represent global developers.

 Much of the article is devoted to the " close playing" of *Detroit: Become Human*. I pause at the conflation of android and slavery and at the invocation of Frederick Douglass without a more careful contextualization of race, gender, and identity in the game. This reader would love to see further contextualization of race, gender, identities, and inclusion of more close playing details across all of the games.

 I do appreciate the "readings" of *Bury, Me My Love, Closed Hands, *and *Chinese Parents*, particularly when the mechanic is to limit player choice, to require the narrative to be directive. More on how these tensions work across and between the games would provide a firmer account of the "ludosis" of the texts. The unpacking of the "ludic grammar of interactive storytelling" in *D:BH* and other games is compelling, though I am ambivalent about (perhaps agnostic regarding) the linking even eliding of play, choice, player agency, and literacy. 

 This respondent questions the immersive fallacy (a la Salen and Zimmerman) and by extension what I have called the interactive fallacy wherein player choice, player agency, the ability to navigate interface, decision trees, and discover different endings belies the permissions, prescriptions, and protocol (cf. Galloway) always-already predetermined by the game's code. That said, I would like to know more about the "conscientious literacy" raised by the article and how that intersects or updates the arguments made by edu-game scholars like James Paul Gee or how that differs from the slide toward gamification: is reading and playing the same thing? Is playing and "authorship" homologous ^1^? The notion of the "text box" as mechanic and readerly/playerful interface is really interesting and could be foregrounded more. Moreover, given the focus on how text and story is deployed by each game, we do not see much *close reading* of the language and narratives themselves. 

 Finally, I appreciate the response to Bogost's polemic and wonder if that contention is too binary, too. I do think the article could incorporate and respond to some more recent work by queer game scholars and scholars of color. As with the concern over immersion, the desire to read and play games as "empathy machines" could be interrogated further via the work by Kishonna Gray, Anna Anthropy, Tanya Pobuda, Bo Ruberg, and others. 

 Overall, the article is thoughtfully written, accessible, and the case studies will be of great use to game scholars, developers, and players.

Is the work clearly and accurately presented and does it cite the current literature?

Partly

If applicable, is the statistical analysis and its interpretation appropriate?

Not applicable

Are all the source data underlying the results available to ensure full reproducibility?

No source data required

Is the study design appropriate and is the work technically sound?

Yes

Are the conclusions drawn adequately supported by the results?

Yes

Are sufficient details of methods and analysis provided to allow replication by others?

Yes

Reviewer Expertise:

Video game studies, analog game studies, queer game studies, literature, pedagogy, "close playing," digital humanities, popular culture

I confirm that I have read this submission and believe that I have an appropriate level of expertise to confirm that it is of an acceptable scientific standard.

## References

[1] : Playing as Making.2018; 10.2307/j.ctv19cwdqv.24 357-368 10.2307/j.ctv19cwdqv.24

# Peer review: Reviewer response for version 1

**DOI:** 10.5256/f1000research.141775.r159900

**Published:** 2023-02-06

**Authors:** Dominic Arsenault

**Affiliations:** 1 Département d'histoire de l'art et d'études cinématographiques, Université de Montréal, Montreal, QC, Canada

The paper provides an analysis of 4 games that were identified by the authors as providing compelling cases that the opposition between ludology and narratology is thin, and perhaps an unproductive way to view video games. The first part of the paper describes the opposing views from proponents of a narrative understanding or approach to video games, and those favoring game mechanics and other terminology or approaches that place the focus of their investigation on the interactivity of games. The main argument is sensible and well-presented.

 The viewpoints, arguments and stances from each camp are adequately summarized. There may be a bit of caricaturing or simplifying of views, which is understandable as much has been written on the subject, but still could be worded a bit better at times. This is a minor perfectible area of the paper.

 To game studies specialists, the time spent in exposing the two conflicting positions of narratology and ludology will probably look like an overly long re-treading of an all-too familiar debate, as the question has been addressed for more than twenty years now. I recommend the authors make it more explicit in the introduction that the part “theorizing ludic narrativity” presents the debate, more particularly to the benefit of readers who may not be aware of the historical development of game studies during the 2000s. I also recommend the author(s) amend the abstract, which claims that “the narrative potential of video games is still being debated”. The debate seems moot, and the most recent writing the author(s) share to that effect is an almost 6-year old text by Bogost in a newspaper.

 These minor issues aside, there is a more substantial hurdle in the paper’s general logic and contribution. Overall, the paper’s argumentation seems like a self-fulfilling prophecy; the time spent in setting up the conflicting positions of narratology and ludology is not well spent since the author(s) have chosen four games that do indeed focus on storytelling rather than mechanics. I appreciate the authors' claim that reading and following the narrative is itself a ludic act / toolkit and vice-versa, but I think the framing context to get to that claim is on an off-axis base. Rewording the first pages would help to streamline the overall thrust of the paper.

 The ”Theorizing ludic narrativity” functions well as a primer for readers unaware of the field of game studies, and the next argumentative move is pertinent in illustrating the ways in which modern games can tackle the issues of narrative progression and narrative comprehension intermingled with the interactive experience.

 The analyses of the four games is a great contribution this paper proposes. The writing is sharp and focused, the points are well-made and the work is illuminating. Overall, the fourth analysis (“Chinese Parents and storytelling of life course”) would benefit from some rewriting and a bit of expansion, if the space allows. It offers less substance than the other three analyses and the writing falls a bit short. The section is descriptively rich (though the familiar language, as in “Once played, it’s totally different.”, contrasts with the previous ones), but I was expecting a more substantial discussion of the implications of these choices and particularities for storytelling with regard to game storytelling. What’s there is good, I was simply expecting a bit more.

 Overall, the paper offers a good contribution through its analyses. It also provides a service to non-game studies scholars for its quick and readable survey of the issues in games and storytelling. Some minor rewrites would be beneficial. A minor rewrite and expansion upon a part is needed for publication. I recommend indexing after these minor revisions are done.

Is the work clearly and accurately presented and does it cite the current literature?

Partly

If applicable, is the statistical analysis and its interpretation appropriate?

Not applicable

Are all the source data underlying the results available to ensure full reproducibility?

No source data required

Is the study design appropriate and is the work technically sound?

Partly

Are the conclusions drawn adequately supported by the results?

Yes

Are sufficient details of methods and analysis provided to allow replication by others?

Yes

Reviewer Expertise:

Game studies, narratology, interactive narrative, history of video games, aesthetics, film studies, animation film

I confirm that I have read this submission and believe that I have an appropriate level of expertise to confirm that it is of an acceptable scientific standard.
